# A systematic review of test accuracy studies evaluating molecular micro-satellite instability testing for the detection of individuals with lynch syndrome

**DOI:** 10.1186/s12885-017-3820-5

**Published:** 2017-12-08

**Authors:** Helen Coelho, Tracey Jones-Hughes, Tristan Snowsill, Simon Briscoe, Nicola Huxley, Ian M. Frayling, Chris Hyde

**Affiliations:** 1 Peninsula Technology Assessment Group (PenTAG), University of Exeter Medical School, South Cloisters, St Lukes Campus, Heavitree Road, Exeter, Devon EX1 2LU UK; 2 Institute of Cancer and Genetics, School of Medicine, Cardiff University, Heath Park, Cardiff, CF14 4XN UK

**Keywords:** Lynch syndrome, HNPCC, Microsatellite instability, Diagnostic testing, Test accuracy, Systematic review

## Abstract

**Background:**

A systematic review was conducted to assess the diagnostic test accuracy of polymerase chain reaction (PCR)-based microsatellite instability (MSI) testing for identifying Lynch syndrome in patients with colorectal cancer (CRC). Unlike previous reviews, this was based on assessing MSI testing against best practice for the reference standard, and included CRC populations that were unselected, age-limited or high-risk for Lynch syndrome.

**Methods:**

Single- and two-gate diagnostic test accuracy studies, or similar, were identified, assessed for inclusion, data extracted and quality appraised by two reviewers according to a pre-specified protocol. Sensitivity of MSI testing was estimated for all included studies. Specificity, likelihood ratios and predictive values were estimated for studies that were not based on high-risk samples. Narrative synthesis was conducted.

**Results:**

Nine study samples were included. When MSI-Low results were considered to be negative, sensitivity estimates ranged from 67% (95% CI 47, 83) to 100% (95% CI 94, 100). Three studies contributed to estimates of both sensitivity and specificity, with specificity ranging from 61% (95% CI 57, 65), to 93% (95% CI 89, 95). Good sensitivity was achieved at the expense of specificity. When MSI-L was considered to be positive (effectively lowering the threshold for a positive index test result) sensitivity increased and specificity decreased. Between-study heterogeneity in both the MSI and reference standard testing, combined with the low number of studies contributing to both sensitivity and specificity estimates, precluded pooling by meta-analysis.

**Conclusions:**

MSI testing is an effective screening test for Lynch syndrome. However, there is significant uncertainty surrounding what balance of sensitivity and specificity will be achieved in clinical practice and how this relates to specific characteristics of the test (such as the panel of markers used or the thresholds used to denote a positive test).

## Background

Lynch syndrome is caused by heritable constitutional pathogenic mutations in the mismatch repair (MMR) genes (*MLH1*, *MSH2*, *MSH6* and *PMS2*) or, rarely, by certain mutations in nearby genes that affect expression of the adjacent MMR gene, (i.e. *EPCAM* and *MSH2,* and *LRRFIP2* and *MLH1*), due to epigenetic silencing caused by promoter methylation [1, 2]. It is responsible for around 2.8% of colorectal cancer (CRC), [3] conveys a high risk of colorectal and endometrial cancer, and increases the risk of other cancers, such as ovarian and gastric cancer [4, 5]. In people with Lynch syndrome, CRC has an earlier onset than CRC in the general population (44 years, compared with 60–65 years respectively) [5, 6]. Currently, the best method for diagnosing Lynch syndrome is comprehensive screening for constitutional mutations in the MMR genes and *EPCAM*, using a combination of (i) DNA sequencing in order to detect point mutations and small insertions and deletions, and (ii) multiplex ligation-dependent probe amplification (MLPA) to detect large structural DNA abnormalities [7].

Patients with CRC can be selected for comprehensive screening for constitutional mutations by first applying other diagnostic tests. Due to the fact that there is a high probability of loss of MMR function in Lynch syndrome cancers, and that tumours which have lost MMR function display microsatellite instability, one such test is microsatellite instability (MSI) testing. This involves polymerase chain reaction (PCR) amplification of DNA markers (using tumour tissue and healthy tissue). The two samples are compared to assess whether abnormal patterns of microsatellite repeats are observed in the tumour DNA. Mono- and dinucleotide markers are the most frequently used with the Bethesda/NCI markers (*BAT-25, BAT-26, D2S123, D5S346, D17S250*) often being used [8]. However, other markers are in use (e.g. *BAT-40, MYCL, MONO-27, NR-21, NR-24*), and it has been argued that the panel should contain at least three mononucleotide markers [9], and thus individual laboratories may develop their own panels [10]. Microsatellite instability is categorised tri-modally (MSI-High, MSI-Low, or MS-Stable) or bi-modally (MSI-positive or negative), according to the proportion of markers demonstrating MSI. When a tri-modal categorisation is initially used, a decision must then be taken as to whether MSI-Low (MSI-L) will then be further categorised as a positive or negative test result.

A previous Health Technology Assessment in England and Wales evaluated the diagnostic test accuracy of MSI for Lynch syndrome in early-onset (aged under 50 years) CRC patients [7]. However, most of the included studies were at risk of bias because the reference standard was not conducted on all participants. Additionally, because this previous review did not include unselected CRC samples, the results may have been subject to spectrum effects and not generalisable to all CRC patients. Furthermore, this previous review [7], and others before it [4, 9, 11], have been obliged to include a wide range of techniques as their reference standard rather than focusing on the primary standard of comprehensive screening for constitutional mutations using a DNA sequencing method combined with MLPA or another appropriate technique to detect large structural DNA abnormalities.

This systematic review was, therefore, conducted to address the need for clearer information regarding the diagnostic test accuracy of PCR-based MSI testing (with or without *BRAF* V600E mutation testing and with or without *MLH1* methylation testing) for identifying Lynch syndrome in patients in the general CRC population. The review was conducted as part of a Diagnostics Assessment Report (DAR) which was commissioned by the National Institute for Health Research (NIHR) Health Technology Assessment Programme in England and Wales to support the National Institute for Health and Care Excellence (NICE) Diagnostics Assessment of molecular testing for Lynch syndrome in people with colorectal cancer [https://www.nice.org.uk/guidance/dg27] [12].

## Methods

The systematic review was undertaken in accordance with a predefined protocol. The protocol for the review (and other reviews in the DAR) can be found at http://www.crd.york.ac.uk/PROSPERO/display_record.asp?ID=CRD42016033879. This review departs from the diagnostic test accuracy review described in the original protocol in that it focuses only on PCR-based MSI testing as the index test, whereas the full review also included immunohistochemistry (IHC) as an index test. However, no studies were found that directly compared MSI testing with IHC and the two index tests were, therefore, reviewed in parallel but evaluated separately.

### Searches

The following bibliographic databases were searched using population terms for Lynch syndrome and intervention terms for MSI or IHC: the Cochrane Database of Systematic Reviews, CENTRAL and HTA [all via the Cochrane library]; MEDLINE, MEDLINE In-Process & Other Non-Indexed Citations, Embase and the Health Management Information Consortium [all via Ovid]; Web of Science [including conference proceedings, via Thomson Reuters]. Search results were limited by date from 2006 to current and to English language publications. The full search strategies are available from the authors.

Four key systematic reviews [4, 7, 9, 11] (and other systematic reviews identified by the bibliographic database searches) were screened in order to source further relevant studies published before 2006 and additional studies published after 2006. These four key systematic reviews [4, 7, 9, 11] were examined prior to the start of this review and were judged to have sufficiently robust searching methods to identify relevant studies published before 2006. Studies which cited the included studies were identified using Scopus (Elsevier). The reference lists of all included studies were screened in order to identify any additional relevant studies.

### Study selection

Titles and abstracts of the studies retrieved from the searches were screened, independently by two reviewers, according to the predefined inclusion criteria specified below. Disagreements between reviewers were resolved by discussion, with arbitration from a third reviewer where necessary. Full texts of included titles/abstracts (from bibliographic database searches, and forward and backward citation chasing), and full texts of studies identified from systematic reviews, were obtained. These were screened in the same way as titles and abstracts.

### Inclusion criteria

Studies had to be single-gate (also known as diagnostic cohort studies) or two-gate (also known as diagnostic case–control studies) diagnostic test accuracy studies (or a variation of one of these designs) [13]. They had to recruit individuals with colorectal cancer and investigate the diagnostic test accuracy of molecular MSI testing (with or without *BRAF* V600E mutation testing and with or without *MLH1* methylation testing). MSI must have been compared with the reference standard, which was constitutional MMR mutation testing (including DNA sequencing of *MLH1*, *MSH2* and *MSH6* and MLPA or another appropriate technique for detecting large genomic abnormalities as a minimum), by providing sufficient data for at least sensitivity to be estimated. Other outcomes (in addition to sensitivity) were: specificity, likelihood ratios (LR+ and LR-), predictive values (PPV and NPV), concordance (with the reference standard), diagnostic yield, and test failure rates. To be included in the review, studies must have been designed for all participants to receive both the index test and reference standard. However, studies recruiting a representative sample of all patients with CRC (including where an age limit was applied), the reference standard may have been applied to all MSI positive-tumours and to a representative (e.g., random) sample of MSI negative-tumours. Abstracts were included if they reported data from an included study that was published in full.

### Data extraction and quality appraisal (risk of bias assessment)

All included studies were given a study identification label: *first-author date*. Where needed for clarity, included studies are identified by their study identification label in the results and discussion sections below. Data extraction and quality appraisal were conducted, for all included studies, by one reviewer and checked by another. Discrepancies were resolved by discussion with the involvement of a third reviewer where necessary. Extracted data included details of the study’s design and methods, participants’ characteristics and study results. Risk of bias in individual studies was assessed according to criteria in Phase 3 of the QUADAS-2 tool [14].

### Analysis and synthesis

The data extracted from the included studies was analysed in STATA 13 (StataCorp LP) using the “diagt” command [15]. For single-gate studies that were not based on high-risk samples (including age-limited population studies), and where data permitted, sensitivity, specificity, LR+, LR-, PPV and NPV, diagnostic yield and concordance with the reference standard (with 95% confidence intervals [CIs]) were calculated. However, for the studies based on high-risk samples, sensitivity (with 95% CI) was calculated (spectrum effects that occur when using a high-risk sample have not been found to lead to significant bias in sensitivity estimates for MSI) [9]. Although not considered an outcome, for illustrative purposes, disease prevalence was also calculated for all included studies, based upon data extracted to 2X2 tables, and representing, therefore, the prevalence in the analysed samples rather than the recruited population. Where extracted data resulted in zero counts in one or more cells, one-sided 95% CIs were calculated.

In primary analyses, MSI-Low was considered as a negative index test result. Unclassified variants (mutations of unknown clinical significance with regards Lynch syndrome), where reported, were considered to be negative reference standard results. The main method of synthesis was narrative.

## Results

**Fig. 1:**
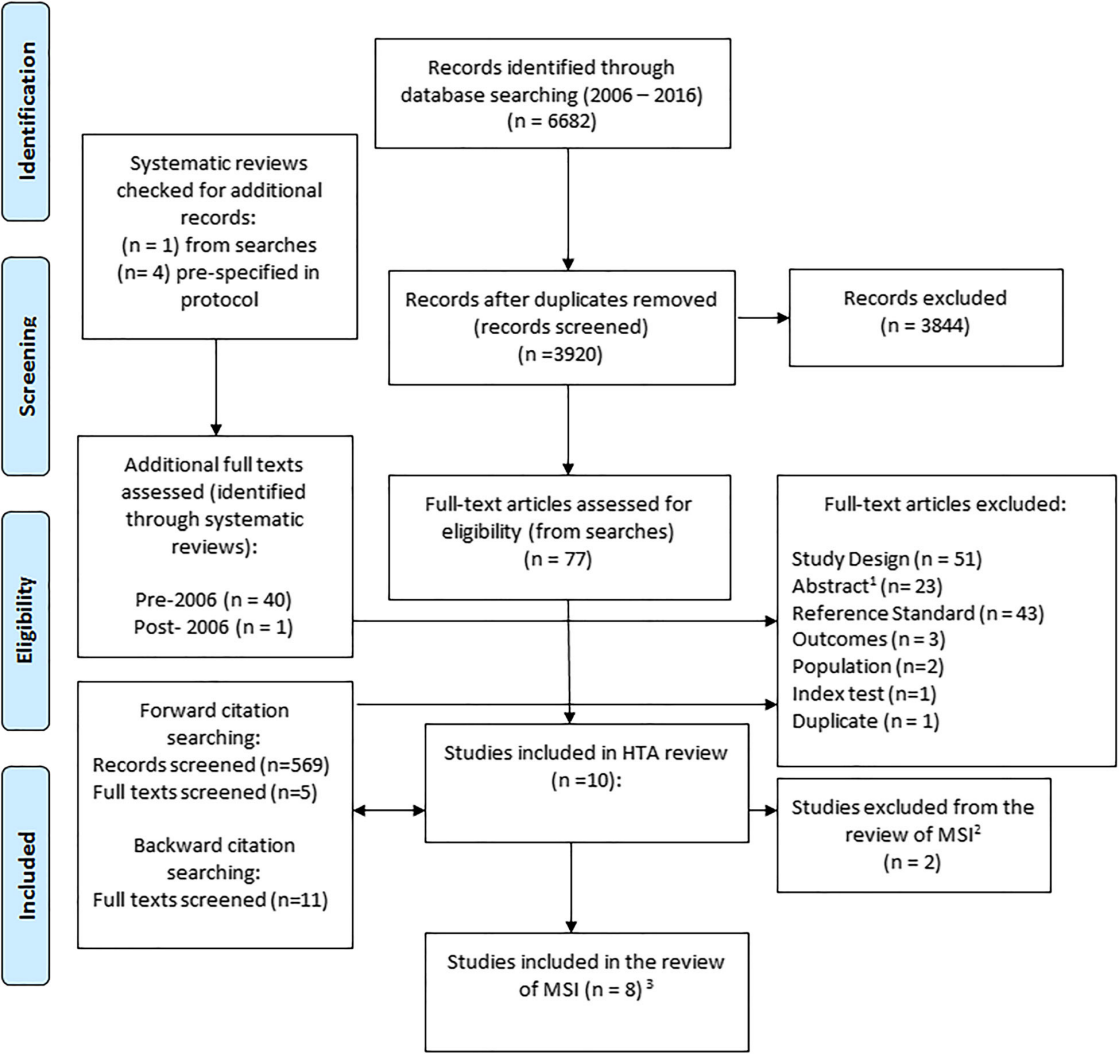
Flow-chart detailing selection of studies. ^1^Abstracts were excluded when they not linked to an included study and did not provide sufficient methodological information to meet the review inclusion criteria or have data extracted. ^2^These studies evaluated IHS and not MSI. ^3^One of these studies included two distinct populations, both of which are included in this review. Although there are eight included studies, there are, therefore, nine included datasets

Ten studies were included in the HTA upon which this review is based (Fig. 1). However, in two of these studies, MSI was not assessed. Therefore, eight studies (*Barnetson 2006, Southey 2005, Poynter 2008, Caldes 2004, Mueller 2009, Overbeek 2007, Shia 2005* and *Hendriks 2003*) [16–23] were eligible for inclusion in this review of MSI testing.

It should be noted that *Poynter 2008* [21] had two distinct samples (a population-based sample and a high-risk sample) and, therefore, had two distinct sets of includable data. These two samples were treated separately and both included in this review.

### Study and participant characteristics

Of the nine study samples included in this review, three report data from a population-based sample, although only *Poynter 2008* recruited an apparently unselected CRC population [21]. The other two studies restricted the population by applying a maximum limit to age of diagnosis: *Barnetson 2006* applied an age limit of <55 years and *Southey 2005* applied a limit of <45 years [16, 23]. All three of these studies used single-gate designs but varied in size with *Barnetson 2006* and *Poynter 2008* recruiting 1259 participants and 1061 participants respectively but *Southey 2005* recruiting only 131 participants [16, 21, 23]. Disease prevalence in the analysed study samples is provided in Table 1 and was similar in *Barnetson 2006* and *Poynter 2008* and higher in *Southey 2005* [16, 21, 23].

**Table 1:** Study and population characteristics

Study	Participant selection and disease prevalence ^a^	Participant characteristics			Reference standard	Microdissection	MSI Panel	MSI thresholds
*Population-based and age-limited single-gate studies*								
Barnetson, 2006	Diagnosed <55yrs of age, consecutive recruitment Disease prevalence 8.5% (95% CI 5.8 to 11.9)	Number:			For all participants: Germ-line DNA obtained from blood leukocytes analysed for MLH1, MSH2, and MSH6 mutations. dHPLC analysis was used for MSH2 and MLH1. Variants noted on chromatography were then sequenced. Mutations were confirmed by re-amplification of an independent sample of DNA and resequencing in both directions. MLH1 and MSH2 were assessed for deletions by MLPA, with products separated on a genetic analyser.	10μm tumour sections; microdissection performed on purified tumour DNA, and control DNA from blood or normal tissue in the section	Bethesda/NCI panel	MSI-H: >1 marker MSI-L: 1 marker MSS: 0 markers
		Recruited		1259				
		Receiving RS		870				
		Receiving MSI		352				
		Gender:						
		Male	53.1%					
		Female	46.9%					
		Age (in years):						
		Non-carrier	48.2 (±6.0)					
		Carrier	42.7 (±7.7)					
		MLH1	38.5 (±8.4)					
		MSH2	43.8 (±6.1)					
		MSH6	49.0 (±3.9)					
		Clinical criteria:						
		AMS II	4.0%					
		RBG	64.0%					
Poynter, 2008^b^	Recruitment through population-based cancer registries (population-based sample), selection process unclear^c^ Disease prevalence 9.2% (95% CI 7.1 to 11.8)	Number:			For all MSI-H or MSI-L probands and in a random sample of 300 MSS population-based probands: Mutations in MSH2 and MLH1 were detected using a combined approach of dHPLC/direct sequencing and MLPA. Direct sequencing was used to detect MSH6 mutations in cases with absent IHC staining of MSH6.	NR	BAT25, BAT26, D5S346, D17S250, BAT40, MYCL, ACTC, Dl 8S55, D1OS197, BAT34C4	MSI-H: ≥30% MSI-L: >0% and <30% MSS: 0%
		Recruited		1061				
		Receiving RS		726				
		Receiving MSI		1061				
		*Gender: NCR*						
		Age (in years): NCR						
		Clinical criteria: NCR						
Southey, 2005	Diagnosed <45yrs of age, random recruitment Disease prevalence 30.5% (95% CI 19.2 to 43.9)	Number:			For all MSI-H or MSI-L probands, those that lacked expression of at least one MMR protein and a random sample of 23 patients selected from those who had tumours that were MS stable and did not lack expression of any MMR protein: MLH1, MSH2, MSH6, and PMS2 genes were screened for germline mutations using sequencing approaches or dHPLC. Confirmation of putative mutations was sought using an independent polymerase chain reaction for direct automated sequencing. MLPA was used to detect large genomic alterations in MLH1 and MSH2 on samples from 10 patients who had tumours lacking at least one MMR protein expression and for which no previous mutation had been identified by sequencing.	5μm tumour sections; microdissection performed on invasive tumour cells from paraffin-embedded archival tumour tissue stained with 1% methyl-green, and normal cells from colonic or lymph node tissue/DNA extracted from peripheral blood lymphocytes	BAT25, BAT26, D2S123, D5S346, D17S250, BAT40, MYB, TGFRII, IGFIIR, BAX	MSI-H: >5 markers MSI-L: 2-5 markers MSS: <2 markers
		Recruited		131				
		Receiving RS		59				
		Receiving MSI		105				
		*Gender:*						
		Male	62.7%					
		Female	37.3%					
		Age (in years):						
		37.1 (range 24 to 42)						
		Clinical criteria:						
		AMS II	9.2%					
		RGB	NR					
*High-risk, single-gate studies*								
Caldes, 2004	HNPCC families selected through a clinic for familial cancer, selection process unclear Disease prevalence 58.6% (95% CI 44.9 to 71.4)	Number:			For all participants: Genomic DNA was isolated from peripheral blood lymphocytes was analysed for MLH1, MSH2 and MSH6. DNA was amplified using PCR and all amplicons were subjected to DGGE or cycle sequencing. The MSI-H cases that were negative for mutations were analysed for genomic deletions in MLH1 and MSH2 by Southern Blotting.	10μm tumour sections; microdissection performed on H&E stained slides with demarked areas containing cancer cells, and corresponding areas on unmarked slides	Bethesda/NCI panel	MSI-H: >1 marker, or 1 marker if BAT26 MSI-L: Not used MSS: 0 markers
		Recruited		58				
		Receiving RS		58				
		Receiving MSI		58				
		*Gender: NR*						
		Age (in years): NR						
		Clinical criteria: NR						
Mueller, 2009	‘Suspected Lynch syndrome’ participants who met Amsterdam criteria, modified Amsterdam criteria, were ‘HNPCC-like’ or met Bethesda criteria, selection process unclear Disease prevalence 58.3% (95% CI 43.2 to 72.4)	Number:			For all participants: Sequencing and MLPA. Limited details provided.	NR	5 and 10 panel markers, no further details provided	NR
		Recruited		48				
		Receiving RS		48				
		Receiving MSI		48				
		*Gender: NR*						
		Age (in years): NR						
		Clinical criteria: NR						
Overbeek, 2007	Families history that fulfilled one of the following criteria: 1) Amsterdam II criteria 2) Bethesda guidelines 3) a history very close to the Bethesda guidelines, selection process unclear Disease prevalence NC	Number:			For all participants: Mutation analysis of MLH1, PMS2, MSH2, and MSH6 was performed in DNA from peripheral blood lymphocytes by a combination of either single-strand conformation polymorphism analysis or DGGE and direct sequence analysis. For the detection of large deletions and duplications in MLH1, MSH2, MSH6, and PMS2, MLPA was used. All deletions and duplications were confirmed by Southern blot analysis or with a specific PCR.	NR	BAT25, BAT26, D2S123, D5S346, D17S250 (BAT40 was also added to the standard set of markers but it is unclear for which participants)	Tumours categorised as positive (>2 Bethesda markers) or negative
		Recruited		NR				
		Receiving RS		NR				
		Receiving MSI		NR				
		*Gender: NR*						
		Age (in years): 40.7 (range 29 to 51)						
		Clinical criteria: NR						
Poynter, 2008^b^	Recruitment through high-risk clinics (clinic-based sample), selection process unclear^c^ Disease prevalence 30.9% (95% CI 23.7 to 38.9)	Number:			For all participants: Mutations in MSH2 and MLH1 were detected using a combined approach of dHPLC/direct sequencing and MLPA. Direct sequencing was used to detect MSH6 mutations in cases with absent IHC staining of MSH6.	NR	BAT25, BAT26, D5S346, D17S250, BAT40, MYCL, ACTC, Dl 8S55, D1OS197, BAT34C4	MSI-H: ≥30% MSI-L: >0% and <30% MSS: 0%
		Recruited		172				
		Receiving RS		152				
		Receiving MSI		172				
		*Gender: NR*						
		Age (in years): NR						
		Clinical criteria: NR						
Shia, 2005	Family history that fulfilled one of the following criteria: I ) Amsterdam I or II criteria 2) a set of relaxed AC three or more colorectal cancers among the first and second-degree relatives of a family that we referred to as ''HNPCC-like,'' and 3) Bethesda criteria, selection process unclear Disease prevalence 49.2 (95% CI 36.1 to 62.3)	Number:			For all participants: Each of the exons of MLH1, MSH2 and MSH6 was amplified by PCR, and heteroduplex analyses were performed using dHPLC. DNA fragments that displayed an abnormal chromatogram were sequenced directly. Cases with tumours that exhibited MSI but in which a point mutation was not detected were analysed for large deletions in MLH1 and MSH2 using a procedure based on the multiplex PCR of short fluorescent fragments.	Microdissection performed on DNA from paraffin-embedded tissue blocks. No further details reported	BAT25, BAT26, D2S123, D17S250, BAT40, PAX6, MYCL1	Tumours categorised as positive (≥30%) or negative
		Recruited		83				
		Receiving RS		83				
		Receiving MSI		Unclear ^d^				
		*Gender:*						
		Male	43.6%					
		Female	56.3%					
		Age (in years):						
		50 (range 23 to 78)						
		Clinical criteria:						
		AMS II	38.2%					
		RBG	8.2%					
*Reference standard positive study*								
Hendriks, 2003	Germline mutation in MLH1, MSH2 or MSH6, selection process unclear Disease prevalence 84.8% (95% CI 68.1 to 94.9)^e^	Number:			For all participants: DGGE or Southern blotting. Limited details provided	Microdissection not specifically reported, paired tumour and normal tissue DNA samples were used	BAT25, BAT26, D2S123, D5S346, D17S250, BAT40, MSH3 and MSH6	MSI-H: >1 Bethesda markers MSI-L: 1 Bethesda marker MSS: 0 Bethesda markers
		Recruited		45				
		Receiving RS		45				
		Receiving MSI		33				
		*Gender:*						
		Male	35.6%					
		Female	40.0%					
		Age (in years):						
		MLH1 48 (range 29 to 90)						
		MSH2 40 (range 23 to 61)						
		MSH6 62 (range 26 to 84)						
		Clinical criteria: NR						

*NC* not calculable, total number of participants with CRC not reported, *NR* Not reported, *NCR* Not clearly reported; ^a^Disease prevalence calculations were based on participants who received both the index test and the reference standard and for whom data were reported. The disease prevalence may, therefore not be an accurate representation of the prevalence in the recruited population, and this is more likely in studies that did not aim to assess the reference standard in all recruited participants; for example, two study samples (the population based sample in Poynter, 2008, and Southey, 2005) did not perform the reference standard in all participants with an MSS result. ^b^Poynter (2008) reports data from two distinct samples, a population-based sample and a high-risk sample; ^c^Although Poynter (2008) reports that ‘some centres recruited all incident cases of CRC while others over­ sampled cases with a family history or early age of onset’ it is not clear whether this applies to the high-risk sample alone or in part to the high-risk sample and in part to the population based sample; ^d^ MSI data are available for 61 participants but it is unclear how many received the test; ^e^Although only reference standard positives were recruited, this included those with an unclassified variant, so when those unclassified variants were considered to be reference standard negatives disease prevalence is 84.8%

The remaining six studies selected participants with CRC who were at high-risk for Lynch syndrome (*Caldes 2004, Hendriks 2003, Mueller 2009, Overbeek 2007*, *Shia 2005* and the other sample in *Poynter 2008*) [17–22]. Five of these studies had a single-gate design [17, 19–22]. The remaining study (*Hendriks 2003*) was a variation on a two-gate study design; although participants with positive reference standard results were recruited, no reference standard negatives were included [18]. We referred to this as a reference standard positive study design. The six high-risk studies varied in size; the largest study was *Poynter 2008* with 172 participants and the smallest study was *Hendriks 2003* with 45 participants [18, 21]. Further details on study and participant characteristics, including disease prevalence in the analysed samples, are given in Table 1.

There was a great deal of between-study variation in both the reference standard and in the MSI testing methods as well as in the reporting of methods. For example, in the studies by *Poynter 2008, Mueller 2009, and Overbeek 2007* microdissection techniques were not reported [19–21]. In addition, none of the population-based studies assessed the same panel of markers, with differences existing in both the number and type of markers, see Table 1. Five studies (*Barnetson 2006, Southey 2005, Poynter 2008, Mueller 2009, Hendriks 2003*) defined tumours tri-modally (i.e. as MSI-H, MSI-L or MSS) [16, 18, 19, 21, 23], two (*Overbeek 2007; Shia 2005*) defined tumours bi-modally (MSI positive or negative) [20, 22], and *Caldes 2004* used a bimodal categorisation but defined tumours as either MSI-H or MSS [17]. The thresholds used to categorise the MSI status of tumours also varied across studies (Table 1), with some studies using positivity of only 20% of markers as the cut-off between MSI-H and MSI-L (*Barnetson 2006*) [16], and others requiring 50% (*Southey 2005*) [23], although different numbers of markers were also used in these two studies.

It should also be mentioned that two of the population-based or age-limited studies (*Poynter 2008, Barnetson 2006*) and three of the high-risk studies (*Caldes 2004, Shia 2005, Hendriks 2003*) report on unclassified variants (i.e. mutations where the association with Lynch syndrome is unclear) [16–18, 21, 22]. Notably, all of the nine studies included in this report predate what is now considered to be the definitive interpretation of MMR gene variants [24]. In this review, therefore, unclassified variants have primarily been considered to be reference standard negative results.

### Risk of bias in individual studies

None of the included studies displayed any evidence to suggest that they were at high-risk of bias (Table 2). It should be noted that an absence of evidence to suggest that the included studies were at high risk of bias does not mean that the studies were free from bias. In fact, none of the studies adequately reported whether MSI was interpreted without knowledge of the reference standard results, or whether the thresholds used to denote a positive MSI result were pre-specified, so it was unclear whether the conduct of the MSI test would have introduced bias. Likewise, for all of the included studies, it was not clear whether the flow and timing of the study would have introduced bias. In all studies except *Hendriks 2003* it was unclear whether the conduct of the reference standard would have introduced bias [18]. Additionally, only *Barnetson 2006* and *Southey 2005* reported sufficient information to determine that participant selection was unlikely to have introduced bias [16, 23].

**Table 2:** Risk of bias assessment using Phase 3 of the QUADAS-2 tool

Domain	Item	Population-based, single-gate			High-risk,single-gate					Other
		Barnetson 2006 [16]	Southey 2005 [23]	Poynter 2008^a^ [21]	Caldes 2004 [17]	Mueller 2009 [19]	Overbeek 2007 [20]	Poynter 2008 ^a^ [21]	Shia 2005 [22]	Hendriks 2003 [18]
Patient selection	Was a consecutive or random sample of patients enrolled?	Y	Y	U	U	U	U	U	U	U
	Was a case-control design avoided?	Y	Y	Y	Y	Y	Y	Y	Y	N^b^
	Did the study avoid inappropriate exclusions?	Y	Y	U	Y	U	U	U	U	Y
	Could the selection of patients have introduced bias?	L	L	U	U	U	U	U	U	U^c^
	Is there concern that the included patients do not match the review question?	L	L	L	L	L	L	L	L	L
Index test	Were the index test results interpreted without knowledge of the results of the reference standard?	U	U	U	U	U	U	U	U	U
	If a threshold was used, was it pre-specified?	U	U	U	U	U	U	U	U	U
	Could the conduct or interpretation of the index test have introduced bias?	U	U	U	U	U	U	U	U	U
	Is there concern that the index test, its conduct, or interpretation differ from the review question?	L	L	L	L	L	L	L	L	L
Reference standard	Is the reference standard likely to correctly classify the target condition?	Y	Y	Y	Y	Y	Y	Y	Y	Y
	Were the reference standard results interpreted without knowledge of the results of the index test?	U	U	U	U	U	U	U	U	Y
	Could the reference standard, its conduct, or its interpretation have introduced bias?	U	U	U	U	U	U	U	U	L
	Is there concern that the target condition as defined by the reference standard does not match the review question?	L	L	L	L	L	L	L	L	L
Flow and timing	Was there an appropriate interval between index test(s) and reference standard?	U	U	U	U	U	U	U	U	U
	Did all patients receive a reference standard?	Y	N	N	Y	Y	Y	Y	Y	Y
	Did patients receive the same reference standard?	Y	N	N	N	U	U	N	N	U
	Were all patients included in the analysis?	N	N	N	N	Y	U	N	U	N
	Could the patient flow have introduced bias?	U	U	U	U	U	U	U	U	U

Notes: ^a^Poynter was assessed twice because data were reported for both a population-based sample and a high-risk sample; ^b^A case-control design was only avoided because there was no control group (half a case control study); ^c^An unbiased estimate of sensitivity (but not specificity) can be ascertained from this study design, however an unclear rating is given because it is not clear if a consecutive or random sample was recruited

Key**:** L = low, N = no, U = unclear, Y = yes

### Sensitivity and specificity

Table 3 gives sensitivity and specificity estimates from primary analyses, where unclassified variants were considered to be index test negative results and MSI-L was considered to be an index test negative result (for studies using a tri-modal distribution of MSI). Only three studies contributed estimates of both sensitivity and specificity (*Barnetson 2006, Southey 2005, Poynter 2008*) [16, 21, 23]. These suggested, in one study (*Poynter 2008*), that good sensitivity (100.0%, 95% CI 93.9100.0) could be achieved at the expense of specificity (61.1%, 95% CI 57.0, 65.1) [21]. Conversely a second study (*Barnetson 2006)* achieved good specificity (92.5%, 95% CI 89.1, 95.2) but at the expense of sensitivity (66.7%, 95% CI 47.2, 82.7) [16]. The third study (*Southey 2005*) had intermediate values of sensitivity (72.2%, 95% CI 46.5, 90.3) and specificity (87.8%, 95% CI 73.8, 95.9) [23]. Although this pattern would be consistent with a threshold effect, it is difficult to establish this because, in addition to using different numbers of unstable markers to denote a positive MSI result, the panel of markers differed between studies (Table 1).

**Table 3:** Sensitivity and specificity estimates (MSI-L categorised as an index test negative result)

Author, year	Sensitivity (%)	Specificity (%)
*Single-gate, population-based samples*		
Poynter, 2008^a^ [21]	100.0 (93.9, 100.0)	61.1 (57.0, 65.1)
Barnetson, 2006 [16]	66.7 (47.2, 82.7)	92.5 (89.1, 95.2)
Southey, 2005 [23]	72.2 (46.5, 90.3)	87.8 (73.8, 95.9)
*Single-gate, high-risk samples*		
Caldes, 2004^b^ [17]	79.4 (62.1, 91.3)	–
Mueller, 2009 [19]	91.3 (72.0, 98.9)	–
Overbeek, 2007^b^ [20]	90.0 (59.6, 98.2)	–
Poynter, 2008^c^ [21]	86.8 (71.9, 95.6)	–
Shia, 2005^b^ [22]	100.0 (85.8, 100.0)	–
*Reference standard positive study*		
Hendriks, 2003 [18]	88.0 (68.8, 97.5)	–

Notes: ^a^Population based sample; ^b^MSI-L not defined; ^c^clinic based sample

The range of sensitivities in the single gate, high risk samples (*Caldes 2004, Mueller 2009, Overbeek 2007*, *Shia 2005* and the other sample in *Poynter 2008*) [17, 19–22] and the reference standard positive study (*Hendriks 2003)* [18] all fell within the range of sensitivities identified in the three single-gate population-based sample studies (*Barnetson 2006*, *Poynter 2008, Southey 2005)* [16, 21, 23].

**Fig. 2:**
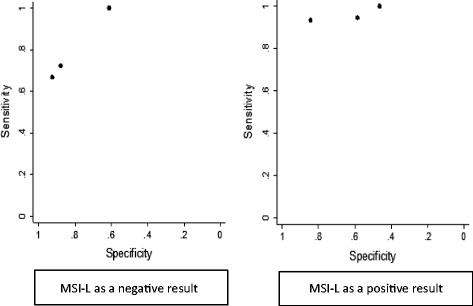
Summary receiver operating characteristic plots for MSI testing

In secondary analyses, when MSI-L was considered to be a positive index test result, sensitivity increased (for the six study samples where a tri-modal distribution of MSI was used) and specificity decreased (for the three population-based samples). This is unsurprising; including MSI-L as an index test positive essentially decreases the threshold for a positive test result. Fig. 2 illustrates this effect for the three population based studies.

Further analyses were also conducted where unclassified variants were considered to be positive reference standard results. Only *Caldes 2004* and *Hendriks 2003* provided sufficient data for these analyses [17, 18]. Both of these studies were based on high-risk populations, so only sensitivity estimates were made, and these were largely unchanged from the primary analyses, most likely because of the low absolute numbers of unclassified variants involved.

Pooling of sensitivity and specificity data was considered but rejected, primarily because of the marked between-study methodological and clinical heterogeneity (e.g. differences in sequencing methods, which genes were tested, in techniques used to test for large genomic deletions and alterations, whether unclassified variants were tested and in the number and nature of the microsatellite makers assessed). Further, as only three studies provided both sensitivity and specificity estimates, this precluded the application of potentially useful test accuracy meta-analytic models such as the hierarchical summary ROC model [25]. Although nine study samples provided estimates of sensitivity, pooling of sensitivity alone is not recommended because of the interdependence with specificity [25–27].

### Secondary outcomes

For the population-based sample in *Poynter 2008* and for the other two studies that recruited population-based samples (*Barnetson 2006 and Southey 2005*) [16, 21, 23], LR+, LR−, PPV, NPV, diagnostic yield and concordance with the reference standard were also calculated. These were primarily estimated based on MSI-L being an index test negative result and these results are given in Table 4. None of the studies reported test failure rates.

**Table 4:** Likelihood ratios, predictive values, yield and concordance (MSI-L categorised as an index test negative result)

Author, year	LR+	LR−	PPV (%)	NPV (%)	Diagnostic yield	Concordance
*Single-gate, population-based samples*						
Poynter, 2008^a^ [21]	2.57 (2.32, 2.85)	0.00 (NE)^b^	20.8 (16.2, 26.0)	100.0 (99.0,100.0)	44.5 (40.6 to 48.5)	64.7 (60.9 to 68.4)
Barnetson, 2006 [16]	8.94 (5.54, 14.20)	0.36 (0.21, 0.60)	45.5 (30.4, 61.2)	96.8 (94.1, 98.4)	12.5 (9.2 to 16.4)	90.3 (86.8 to 93.2)
Southey, 2005 [23]	5.92 (2.48, 14.10)	0.32 (0.15, 0.67)	72.2 (46.5, 90.3)	87.8 (73.8, 95.9)	30.5 (19.2 to 43.9)	83.0 (71.0 to 91.6)

Notes: ^a^Population based sample; ^b^Not estimable

## Discussion

This systematic review was conducted by an independent, experienced research team working to a pre-specified protocol. It is notable that, of the nine study samples included in this review, only one (*Poynter 2008*) appeared to recruit an unselected CRC population, although even in this study it was not clear how the participants were selected [21]. This paucity of large, unselected, population-based studies is unsurprising; it is costly to provide all participants with both the index test and the reference standard. However, it would be methodologically acceptable to perform the reference standard on a random sample of index test negatives (and all index test positives), and this would decrease costs.

Despite improvements in reference standard techniques and, therefore, stricter definitions of the reference standard in this review compared with previous reviews, a similar range of test accuracy estimates were found [4, 7, 9, 11]. Indeed, across all nine study samples, sensitivity ranged from 66.7% (95% CI 47.2, 82.7) to 100.0% (95% CI 93.9, 100.0) in primary analyses. This is broadly in line with previous reviews [9, 11]. In the three population-based studies identified in this review, specificity estimates ranged from 61.1% (95% CI 57.0, 65.1) in *Poynter 2008* to 92.5% (95% CI 89.1, 95.2) in *Barnetson 2006* [16, 21].

Across all included studies, sensitivity estimates did not appear to be greatly impacted by the type of population; the estimates generated from the high-risk samples [17–22] were not obviously dissimilar from the population-based samples [16, 21, 23]. Similar results have been noted in a previous review, where it was suggested that spectrum bias may not be an issue for estimating sensitivity of MSI testing [9]. There is, however, good evidence that MSI prevalence in sporadic cancers increases with age [28], which could result in increased false positive results in older populations and, therefore, impact upon specificity estimates [29].

There are many possible explanations for the between-study differences in the sensitivity and specificity estimates. It is clear from the current review that MSI testing is not a universally standard procedure; differences exist in the way in which these tests are performed (including different thresholds used in each study to denote an MSI positive result, in the specific genetic testing procedures used for the reference standard, and in the different panel of MSI markers used in each study). These differences may impact upon the number of false negatives and false positives generated, and therefore the sensitivity and specificity of the test. As there is always a trade-off between sensitivity and specificity, and this would likely be influenced by differences in the MSI-testing procedures, it is important to consider whether sensitivity should be maximized at the expense of specificity or vice versa. There was not a sufficient body of high-quality evidence to conduct meta-regression, which could make it possible to predict the balance of sensitivity and specificity for a given set of test characteristics, but it is unavoidable that using a lower threshold (e.g. including MSI-L as a positive test result) would increase sensitivity at the expense of specificity.

Due to the fact that an MSI test is a triage test rather than a definitive diagnostic test, and assuming an aim of maximising the number of individuals with Lynch syndrome who eventually receive a correct diagnosis, it would be preferable to maximize sensitivity (i.e. try to minimize false negative MSI results at the expense of false positives). False positive results from MSI testing are likely to be corrected by subsequent testing, while false negative results are unlikely to be corrected until there is another cancer in the individual or their family. However, false positive results can still have direct and undesirable effects on health (e.g., anxiety related to genetic counselling and genetic testing) and also on the health service (due to the cost of unnecessary testing), and in some patients genetic testing may not be conclusive, which can lead to difficulties in identifying appropriate clinical management [24]. Indeed, it is virtually impossible to estimate the relative harms of false negative and false positive results without some form of evidence synthesis approach, such as decision modelling [30].

One of the key strengths of the current systematic review is that it did include studies from a range of populations and using a range of different MSI testing strategies (with different panels of markers and thresholds). Diagnostic test accuracy estimates are provided for each of the included studies rather than providing pooled estimates that may not apply to a particular population or testing strategy. For clinicians, patients, academics and anyone else wanting estimates of the sensitivity and specificity of MSI-testing for identifying pathogenic Lynch syndrome mutations, it may be prudent to look at the estimates from studies whose samples and testing methods are most similar to the population of interest rather than using pooled estimates from heterogeneous studies. Unfortunately, the paucity of similar studies precluded the statistical investigation of factors impacting sensitivity and specificity estimates (e.g., markers, thresholds). If future studies and analyses accurately quantify the trade-off between sensitivity and specificity according to test characteristics, a decision modelling approach could be used to select the appropriate characteristics to maximise the desired objective (e.g., cost-effectiveness).

## Conclusion

MSI testing is an effective screening test for Lynch syndrome. However, there is a paucity of studies that evaluate test accuracy in unselected, population-based samples. In addition, the studies that were identified in this review displayed heterogeneity in both the MSI and reference standard testing methods. As such, there is significant uncertainty surrounding what balance of sensitivity and specificity will be achieved in clinical practice and how this relates to specific characteristics of the test (such as the panel of markers used or the thresholds used to denote a positive test).

## Abbreviations

CI: Confidence Intervals
CRC: Colorectal Cancer
DAR: Diagnostics Assessment Report
IHC: Immunohistochemistry
LR +: Positive Likelihood Ratios
LR-: Negative Likelihood Ratios
MLPA: Multiplex Ligation-Dependent Probe Amplification
MMR: Mismatch Repair
MSI: Microsatellite Instability
NICE: National Institute For Health And Care Excellence
NIHR: National Institute For Health Research
NPV: Negative Predictive Values
PCR: Polymerase Chain Reaction
PPV: Positive Predictive Values

## References

[1] Morak M, Koehler U, Schackert HK, Steinke V, Royer-Pokora B, Schulmann K, Kloor M, Hochter W, Weingart J, Keiling C (2011). Biallelic MLH1 SNP cDNA expression or constitutional promoter methylation can hide genomic rearrangements causing lynch syndrome. J Med Genet.

[2] Niessen RC, Hofstra RM, Westers H, Ligtenberg MJ, Kooi K, Jager PO, de Groote ML, Dijkhuizen T, Olderode-Berends MJ, Hollema H (2009). Germline hypermethylation of MLH1 and EPCAM deletions are a frequent cause of lynch syndrome. Genes Chromosomes Cancer.

[3] Hampel H, de la Chapelle A (2013). How do we approach the goal of identifying everybody with lynch syndrome?. Familial Cancer.

[4] Vasen HF, Blanco I, Aktan-Collan K, Gopie JP, Alonso A, Aretz S, Bernstein I, Bertario L, Burn J, Capella G (2013). Revised guidelines for the clinical management of lynch syndrome (HNPCC): recommendations by a group of European experts. Gut.

[5] Møller P, Seppälä T, Bernstein I, Holinski-Feder E, Sala P, Evans DG, Lindblom A, Macrae F, Blanco I, Sijmons R *et al*: Cancer incidence and survival in Lynch syndrome patients receiving colonoscopic and gynaecological surveillance: first report from the prospective Lynch syndrome database. *Gut* 2015, Online first (9 December 2015). 10.1136/gutjnl-2015-309675 PMC5534760 26657901

[6] Munoz. Hereditary Colorectal Cancer. 2017. [http://emedicine.medscape.com/article/188613-overview#a5].

[7] Snowsill T, Huxley N, Hoyle M, Jones-Hughes T, Coelho H, Cooper C, Frayling I, Hyde C (2014). A systematic review and economic evaluation of diagnostic strategies for lynch syndrome. Health Technol Assess.

[8] Umar A, Boland CR, Terdiman JP, Syngal S, de la Chapelle A, Ruschoff J, Fishel R, Lindor NM, Burgart LJ, Hamelin R (2004). Revised Bethesda guidelines for hereditary nonpolyposis colorectal cancer (lynch syndrome) and microsatellite instability. J Natl Cancer Inst.

[9] Palomaki GE, McClain MR, Melillo S, Hampel HL, Thibodeau SN (2009). EGAPP supplementary evidence review: DNA testing strategies aimed at reducing morbidity and mortality from lynch syndrome. Genet. Med..

[10] Frayling I, Berry I, Wallace A, Payne S, Norbury G: ACGS Best practice guidelines for genetic testing and diagnosis of Lynch syndrome. Association for Clinical Genetic Science 2016, ://www.acgs.uk.com/media/998715/ls_bpg_approved.pdf.

[11] Bonis PA, Trikalinos TA, Chung M, Chew P, Ip S, DeVine DA, Lau J, Tufts-New England Medical Center. Evidence-based practice center, United States. Agency for Healthcare Research and Quality: hereditary nonpolyposis colorectal cancer : diagnostic strategies and their implications. Rockville, MD: U.S. Dept. of Health and Human Services, Public Health Service, Agency for Healthcare Research and Quality; 2007.

[12] Peninsula Technology Assessment Group (PenTAG): Molecular testing for Lynch syndrome in people with colorectal cancer [available from https://www.nice.org.uk/guidance/GID-DG10001/documents/final-protocol-2]. In*.*; 2016. 10.3310/hta21510 PMC5611555 28895526

[13] Rutjes AW, Reitsma JB, Vandenbroucke JP, Glas AS, Bossuyt PM (2005). Case-control and two-gate designs in diagnostic accuracy studies. Clin Chem.

[14] Whiting PF, Rutjes AW, Westwood ME, Mallett S, Deeks JJ, Reitsma JB, Leeflang MM, Sterne JA, Bossuyt PM (2011). Group Q-: QUADAS-2: a revised tool for the quality assessment of diagnostic accuracy studies. Ann Intern Med.

[15] Seed PA, T: Summary statistics for diagnostoc tests. Stata Technical Bulletin 2001, 10(59).

[16] Barnetson RA, Tenesa A, Farrington SM, Nicholl ID, Cetnarskyj R, Porteous ME, Campbell H, Dunlop MG (2006). Identification and survival of carriers of mutations in DNA mismatch-repair genes in colon cancer. N Engl J Med.

[17] Caldes T, Godino J, Sanchez A, Corbacho C, De La Hoya M, Asenjo JL, Saez C, Sanz J, Benito M, Cajal SRY (2004). Immunohistochemistry and microsatellite instability testing for selecting MLH1, MSH2 and MSH6 mutation carriers in hereditary non-polyposis colorectal cancer. Oncol Rep.

[18] Hendriks Y, Franken P, Dierssen JW, de Leeuw W, Wijnen J, Dreef E, Tops C, Breuning M, Bröcker-Vriends A, Vasen H (2003). Conventional and tissue microarray immunohistochemical expression analysis of mismatch repair in hereditary colorectal tumors. Am J Pathol.

[19] Mueller J, Gazzoli I, Bandipalliam P, Garber JE, Syngal S, Kolodner RD (2009). Comprehensive molecular analysis of mismatch repair gene defects in suspected lynch syndrome (hereditary nonpolyposis colorectal cancer) cases. Cancer Res.

[20] Overbeek L, Kets C, Hebeda K, Bodmer D, Van Der Looij E, Willems R, Goossens M, Arts N, Brunner H, Van Krieken J (2007). Patients with an unexplained microsatellite instable tumour have a low risk of familial cancer. Br J Cancer.

[21] Poynter JN, Siegmund KD, Weisenberger DJ, Long TI, Thibodeau SN, Lindor N, Young J, Jenkins MA, Hopper JL, Baron JA (2008). Molecular characterization of MSI-H colorectal cancer by MLHI promoter methylation, immunohistochemistry, and mismatch repair germline mutation screening. Cancer Epidemiol Biomark Prev.

[22] Shia J, Klimstra DS, Nafa K, Offit K, Guillem JG, Markowitz AJ, Gerald WL, Ellis NA (2005). Value of immunohistochemical detection of DNA mismatch repair proteins in predicting germline mutation in hereditary colorectal neoplasms. Am J Surg Pathol.

[23] Southey MC, Jenkins MA, Mead L, Whitty J, Trivett M, Tesoriero AA, Smith LD, Jennings K, Grubb G, Royce SG (2005). Use of molecular tumor characteristics to prioritize mismatch repair gene testing in early-onset colorectal cancer. J Clin Oncol.

[24] Thompson BA, Spurdle AB, Plazzer J-P, Greenblatt MS, Akagi K, Al-Mulla F, Bapat B, Bernstein I, Capella G, den Dunnen JT (2014). Application of a 5-tiered scheme for standardized classification of 2,360 unique mismatch repair gene variants in the InSiGHT locus-specific database. Nat Genet.

[25] Rutter CM, Gatsonis CA (2001). A hierarchical regression approach to meta-analysis of diagnostic test accuracy evaluations. Stat Med.

[26] Moses LE, Shapiro D, Littenberg B (1993). Combining independent studies of a diagnostic test into a summary ROC curve: data-analytic approaches and some additional considerations. Stat Med.

[27] Reitsma JB, Glas AS, Rutjes AW, Scholten RJ, Bossuyt PM, Zwinderman AH (2005). Bivariate analysis of sensitivity and specificity produces informative summary measures in diagnostic reviews. J Clin Epidemiol.

[28] Sie AS, Mensenkamp AR, Adang EM, Ligtenberg MJ, Hoogerbrugge N (2014). Fourfold increased detection of lynch syndrome by raising age limit for tumour genetic testing from 50 to 70 years is cost-effective. Ann Oncol.

[29] van Lier MG, Leenen CH, Wagner A, Ramsoekh D, Dubbink HJ, van den Ouweland AM, Westenend PJ, de Graaf EJ, Wolters LM, Vrijland WW (2012). Yield of routine molecular analyses in colorectal cancer patients </=70 years to detect underlying lynch syndrome. J Pathol.

[30] Ferrante di Ruffano L, Hyde CJ, McCaffery KJ, Bossuyt PM, Deeks JJ (2012). Assessing the value of diagnostic tests: a framework for designing and evaluating trials. BMJ.

